# 103. Respiratory syncytial virus (RSV) sequence variation and related clinical phenotypes in young children across six respiratory seasons

**DOI:** 10.1093/ofid/ofaf695.038

**Published:** 2026-01-11

**Authors:** Diego R Hijano, Helena Brenes-Chacon, Marie Wehenkel, Bart G Jones, Dolores Acuna, Lei Li, Gang Wu, Jessica Brazelton, Randall Hayden, Octavio Ramilo, Asuncion Mejias

**Affiliations:** St. Jude Children's Research Hospital, Memphis, TN; St. Jude Children's Research Hospital, Memphis, TN; St. Jude Children's Research Hospital, Memphis, TN; St. Jude, Memphis, Tennessee; National Scientific and Technical Research Council (CONICET), Argentina. Department of Infectious Diseases, St Jude Children’s Research Hospital, I Memphis, Tennessee, United States., La Plata, Buenos Aires, Argentina; St. Jude Children's Research Hospital, Memphis, TN; St. Jude Children's Research Hospital, Memphis, TN; St. Jude Children's Research Hospital, Memphis, TN; St. Jude Children's Research Hospital, Memphis, TN; St. Jude Children's Research Hospital, Memphis, TN; St Jude Children's Research Hospital, Memphis, TN

## Abstract

**Background:**

Implementation of RSV whole genome sequencing has identified several lineages and RSV mutants circulating worldwide, but their impact on children’s clinical phenotype is unknown. We evaluated RSV genetic variation and related children’s clinical phenotypes before the introduction of new RSV monoclonals in the USA.

**Figure:**
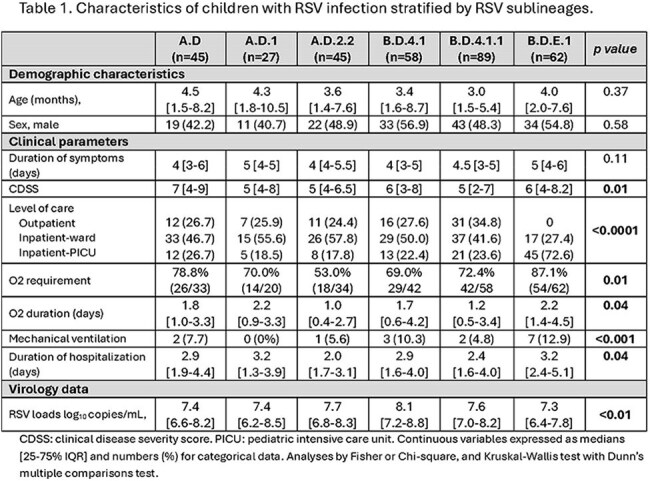

**Figure 1:**
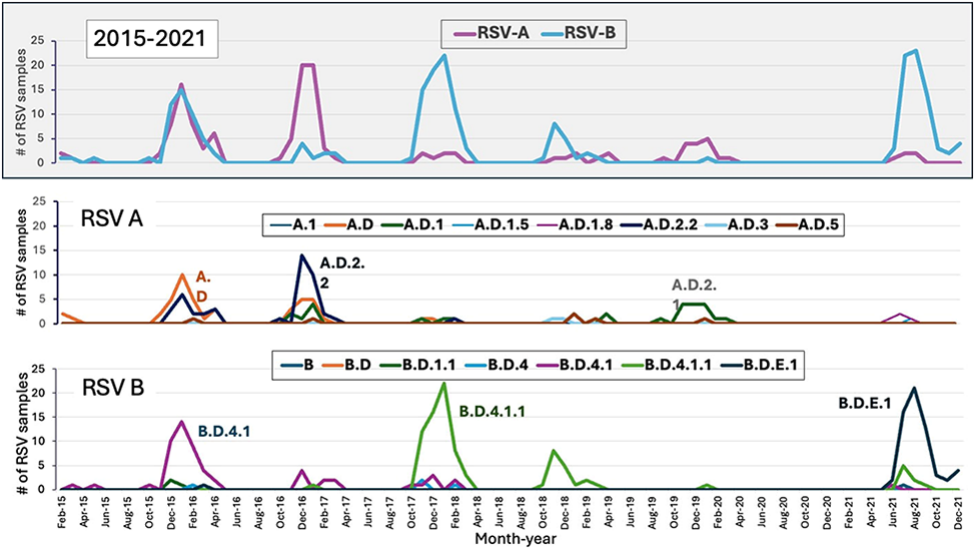
RSV A and RSV B sublineages circulating between 2015-2021

**Methods:**

A prospective, single center study was performed using a convenience sample of previously healthy children < 2 years hospitalized or evaluated as outpatients with RSV infection during 6 seasons (2015-21). Nasopharyngeal swabs were collected at enrollment, RSV loads measured by quantitative RT-PCR, and whole genome sequence analyzed with a computational pipeline referencing the RSV Genotyping Consensus Consortium. Clinical outcomes were analyzed according to the main RSV A and B sublineages.

**Figure:**
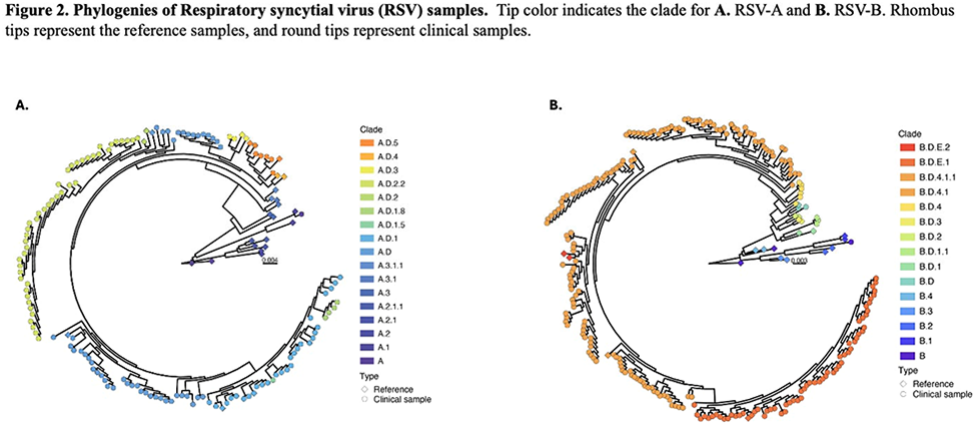

**Figure:**
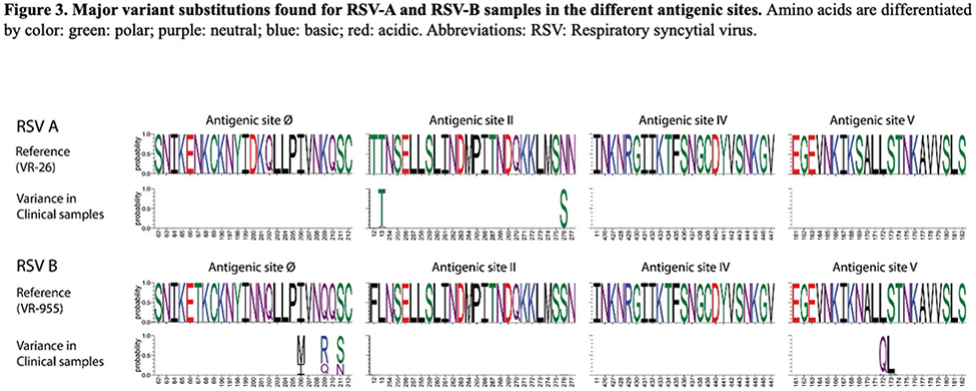

**Results:**

Samples from 349 children were analyzed (131 RSV-A (38%), 218 RSV-B (62%)). Children’s median [IQR] age was 3.5 [1.6-8.0] months. Phylogenetic analysis identified the co-circulation of eight RSV-A sublineages primarily derived from A.D. (n=45; 34%), A.D.2.2 (n=45; 34%), and A.D.1 (n=27, 21%). Among RSV B infections we identified 7 sublineages mostly derived from B.D.4.1.1 (n=89; 41%), B.D.E.1 (n=62; 28%), and B.D.4.1 (n=58; 27%). RSV A.D, A.D.2.2, B.D.4.1, and B.D.4.1.1 predominated pre-pandemic, while B.D.E.1 predominated during 2021 (Fig 1, 2). While RSV B isolates exhibited several clinically important mutations in antigenic sites V and Ø, most RSV A strains showed no mutations. When mutations in RSV-A strains were present, they were limited to antigenic site II (Fig 3). Among the most prevalent sublineages, children infected with RSV B.D.E.1 had significantly higher rates of hospitalization, O2 requirement, PICU admission, longer hospitalizations, and lower RSV loads (Table 1).

**Conclusion:**

Different RSV-A and B sublineages circulated from 2015-21 with variable distributions during respiratory seasons and distinct impact on clinical disease severity. Mutations, although infrequent, were more prevalent in RSV-B strains, including in sites Ø and V. While the clinical relevance of these mutations needs further study, these results underscore the importance of continuous RSV sequence-based surveillance.

**Disclosures:**

Octavio Ramilo, MD, Gates Foundation: Grant/Research Support|Merck, Sharpe & Dohme: Advisor/Consultant|Merck, Sharpe & Dohme: Grant/Research Support|Merck, Sharpe & Dohme: Honoraria|Moderna: Advisor/Consultant|NIH/NIAID: Board Member|NIH/NIAID: Grant/Research Support|Pfizer, Inc.: Advisor/Consultant|Pfizer, Inc.: Honoraria|Sanofi Pasteur LLC: Advisor/Consultant|Sanofi Pasteur LLC: Honoraria

